# Predicting high-risk endometrioid carcinomas using proteins

**DOI:** 10.18632/oncotarget.24803

**Published:** 2018-04-13

**Authors:** Di Du, Wencai Ma, Melinda S. Yates, Tao Chen, Karen H. Lu, Yiling Lu, John N. Weinstein, Russell R. Broaddus, Gordon B. Mills, Yuexin Liu

**Affiliations:** ^1^ Department of Bioinformatics and Computational Biology, The University of Texas MD Anderson Cancer Center, Houston, Texas, USA; ^2^ Department of Gynecologic Oncology and Reproductive Medicine, The University of Texas MD Anderson Cancer Center, Houston, Texas, USA; ^3^ Endoscopy Center and Endoscopy Research Institute, Zhongshan Hospital of Fudan University, Shanghai, China; ^4^ Department of Systems Biology, The University of Texas MD Anderson Cancer Center, Houston, Texas, USA; ^5^ Department of Pathology, The University of Texas MD Anderson Cancer Center, Houston, Texas, USA

**Keywords:** RPPA, stage, protein, biomarker, endometrioid carcinoma

## Abstract

**Background:**

The lethality of endometrioid endometrial cancer (EEC) is primarily attributable to advanced-stage diseases. We sought to develop a biomarker model that predicts EEC surgical stage at the time of clinical diagnosis.

**Results:**

PSES was significantly correlated with surgical stage in the TCGA cohort (*P* < 0.0001) and in the validation cohort (*P* = 0.0003). Even among grade 1 or 2 tumors, PSES was significantly higher in advanced than in early stage tumors in both the TCGA (*P* = 0.005) and MD Anderson Cancer Center (MDACC) (*P* = 0.006) cohorts. Patients with positive PSES score had significantly shorter progression-free survival than those with negative PSES in the TCGA (hazard ratio [HR], 2.033; 95% CI, 1.031 to 3.809; *P* = 0.04) and validation (HR, 3.306; 95% CI, 1.836 to 9.436; *P* = 0.0007) cohorts. The ErbB signaling pathway was most significantly enriched in the PSES proteins and downregulated in advanced stage tumors.

**Methods:**

Using reverse-phase protein array expression profiles of 170 antibodies for 210 EEC cases from TCGA, we constructed a Protein Scoring of EEC Staging (PSES) scheme comprising 6 proteins (3 of them phosphorylated) for surgical stage prediction. We validated and evaluated its diagnostic potential in an independent cohort of 184 EEC cases obtained at MDACC using receiver operating characteristic curve analyses. Kaplan-Meier survival analysis was used to examine the association of PSES score with patient outcome, and Ingenuity pathway analysis was used to identify relevant signaling pathways. Two-sided statistical tests were used.

**Conclusions:**

PSES may provide clinically useful prediction of high-risk tumors and offer new insights into tumor biology in EEC.

## INTRODUCTION

Endometrial carcinoma is the most common gynecologic malignancy; over 10,470 deaths from uterine corpus cancer were expected in the United States in 2016 [1], an approximately three-fold increase over the past 25 years. The lethality of endometrial cancer is primarily correlated with stage III or IV (hereafter referred to as advanced stage) disease [2]. Typically, a five-year survival rate of 83%–97% is achieved for localized disease (stage I/II, hereafter referred to as early stage), in contrast to 43%-67% for stage III disease and only 13%–25% for stage IV disease [3]. Patients with advanced stage disease are at an increased risk of recurrence and are typically treated with postoperative radiotherapy and/or chemotherapy; however, this is rarely curable [4]. Therefore, early stratification of endometrial carcinomas into surgical staging categories from tissue biopsies will help gynecologic oncologists more accurately choose which women should have extensive surgical staging procedures or receive systemic adjuvant therapy, and thus represents a key to reducing the morbidity and mortality associated with endometrial cancer.

Non-endometrioid carcinoma is known a priori to be clinically aggressive and can be readily segregated from its endometrioid counterpart at the time of diagnosis [5] because these two entities exhibit striking difference in histopathological (microscopic) appearance and molecular characteristics such as *TP53* mutation, [6] *CTNNB1* mutation [7], protein levels of estrogen receptor/progesterone receptor (ER/PR) [8], and gene expression profiling [9]. Endometrioid-type endometrial cancer (EEC), accounting for approximately 70 to 80% of endometrial cancer cases, is the focus of the current study. EEC is pathologically staged with the International Federation of Gynecology and Obstetrics (FIGO) system [2]. However, molecular characteristics have not been validated to help predict operative staging of EEC patients at the time of clinical diagnosis [10, 11]. Since molecular abnormalities predispose the manifestation of microscopic appearance and drive tumor progression, molecular biomarkers not only provide an opportunity for early detection but also offer the ability to direct therapeutic strategies; both are distinctive from the current microscopic analysis.

Cellular proteins are responsible for functional diversity because a vast array of regulatory or metabolic processes occur at the protein level; these cannot be adequately predicted from DNA or RNA data. The reverse-phase protein array (RPPA) platform allows identification of proteins for use as therapeutic intervention or as markers for the classification of cancer [12–14]. In this study, we analyzed the protein expression profiles of over 200 EEC cases with clinicopathologic characteristics obtained from The Cancer Genome Atlas (TCGA) and developed a Protein Scoring of EEC Staging (PSES) scheme that we further validated in an independent MD Anderson Cancer Center (MDACC) cohort comprising an additional 184 EEC cases. Prognostic and biological significance of PSES was further investigated. Our work demonstrates the potential of clinical stratification and novel therapeutic targets.

## RESULTS

### Patient characteristics

Clinicopathologic characteristics of patients included in the TCGA and MDACC cohorts are described and compared in Table 1. All patients were endometrial cancer with endometrioid histology. The two cohorts had a comparable percentage of advanced stage disease (TCGA, 21.4%; MDACC, 26.1%), but there were significantly more grade 3 tumors in the TCGA cohort than in the MDACC cohort (39.0% versus 19.3%, *P* < 0.0001, Fisher's exact test). No statistical difference was observed between these two cohorts for patient age, death or recurrence events, or patient outcome, including overall survival (OS) and progression-free survival (PFS) (Supplementary Figure 1).

**Table 1:** Clinicopathologic characteristics of EEC patients in the training (TCGA) and validation (MDACC) cohorts^*^

	TCGA (*n* = 210)	MDACC (*n* = 184)	*P* ^†^
**Age**			
Mean, years [SD]	60.3 [11.3]	59.8 [11.6]	0.3934^ξ^
Range	30.5–87.5	27.2–84.7	
**FIGO Stage**			
I/II	165 (78.6)	136 (73.9)	
III/IV	45 (21.4)	48 (26.1)	0.2870^¶^
**Histological Grade**			
1/2	128 (61.0)	146 (80.7)	
3	82 (39.0)	35 (19.3)	< 0.0001^¶^
Unknown	0	3	
**Vital Status**			
Living	190 (90.5)	158 (87.8)	
Deceased	20 (9.5)	22 (12.2)	0.4162^¶^
Unknown	0	4	
**Recurrent Disease**			
No	162 (81.0)	123 (77.8)	
Yes	38 (19.0)	35 (22.2)	0.5079^¶^
Unknown	10	26	
**MSI Status**			
MSI^‡^	91 (43.3)	NA	
MSS	119 (56.7)	NA	

Abbreviations: TCGA, The Cancer Genome Atlas; MDACC, MD Anderson Cancer Center; SD, standard deviation; FIGO, International Federation of Gynecology and Obstetrics; MSI, microsatellite instability; MSS, microsatellite stable; NA, not applicable.

^*^Values are reported as No. (%). Missing values are excluded from percentage calculation and statistical test.

^†^Statistical comparison of clinicopathological features between patients in the TCGA cohort and those in the MDACC cohort.

^ξ^Mann–Whitney test.

^¶^Fisher's exact test.

^‡^Including both MSI-H and MSI-L.

### Generation of PSES

**Figure 1:**
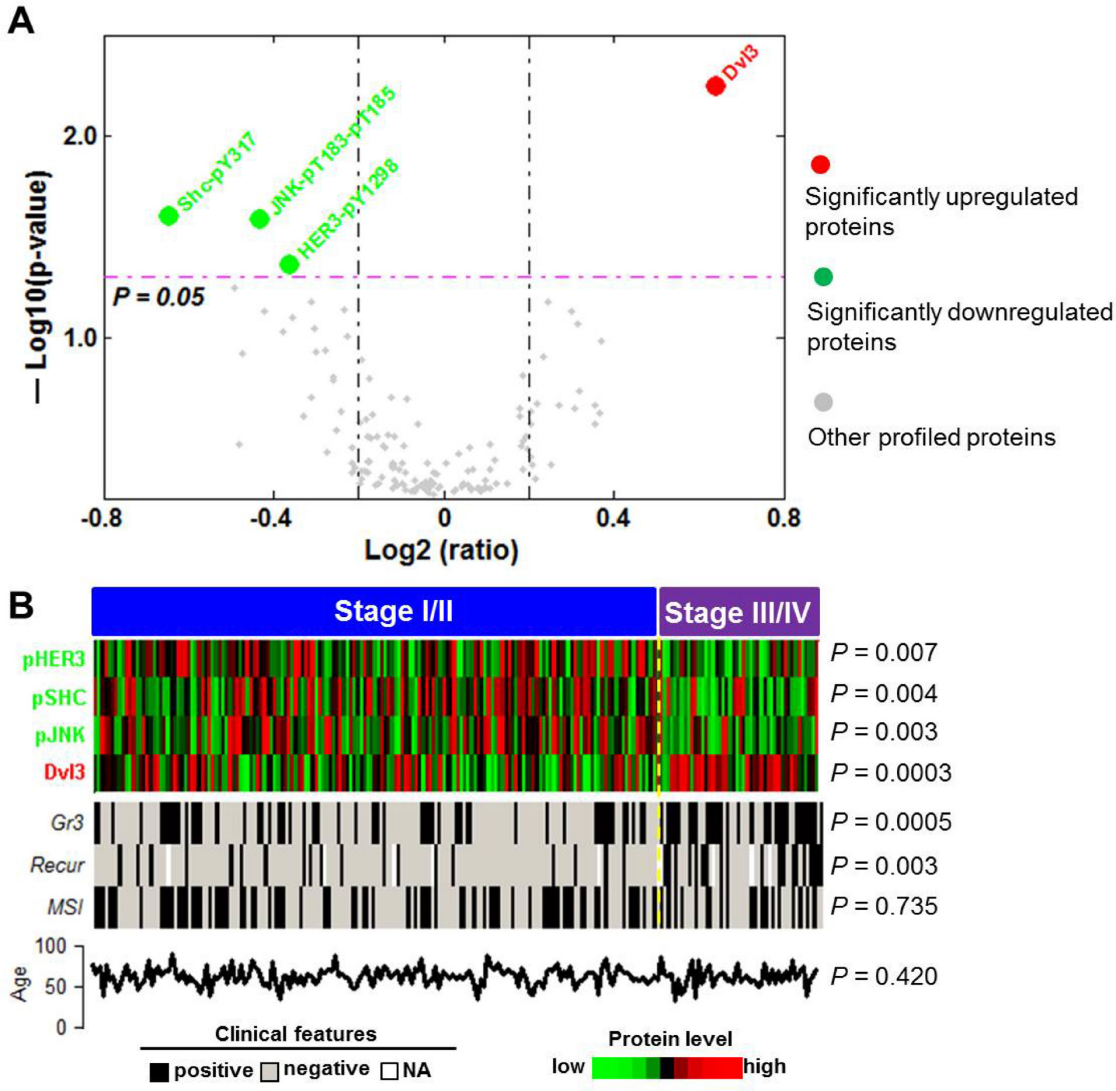
Identification of protein markers used for construction of PSES (**A**) Volcano plot shows the protein expression difference (in terms of log2 ratio) and the corresponding statistical tests (in terms of -log10 (*P* value)) between Stage III/IV tumors and Stage I/II tumors. Values were averaged from those calculated from the 500 different subsets randomly selected from the TCGA cohort. Four proteins exhibited a statistically significant difference. The dashed pink line indicates the *P* value cutoff of 0.05. (**B**) Relative expression levels of the four proteins and clinicopathologic characteristics for each individual patient in the entire TCGA cohort (*n* = 210). The *P* values show the comparison between the early stage patients vs advanced stage patients and are calculated from the entire TCGA cohort.

Using the random partitioning method and an average *P* value cutoff of 0.05 (see Methods for details), we identified four proteins or phosphoproteins that were significantly associated with advanced stage disease: one upregulated protein (Dvl3) and three downregulated phosphoproteins, Shc-pY317, JNK-pT183-pT185, and HER3-pY1298 (hereafter referred to as pSHC, pJNK, and pHER3, respectively) (Figure 1A). The relative expression levels of these four proteins for each individual across the entire TCGA cohort were also depicted (Figure 1B). Consistent with the results rendered by the random approach, the four proteins were significantly differentially expressed between early and advanced stage tumors in the entire TCGA cohort. Moreover, the advanced stage tumors were significantly associated with grade 3 tumors and recurrent events, but not with MSI status or patient age (Figure 1B). Given that EEC tumors are typically characterized by the expression levels of estrogen receptor alpha (ER) and progesterone receptor (PR), we included these two proteins in the predictive model to account for baseline fluctuation on other clinicopathological variables such as age, grade, and recurrence status. A similar approach of inclusion of endogenous hormone levels in risk prediction models for postmenopausal breast cancer was applied previously in improving the ability to identify high-risk women [15]. Thus, total six proteins were used to compose the predictive model, and we termed this predictive protein set PSES (Protein Scoring of EEC Staging). The predictive model, expressed as PSES score, was defined as a linear combination of the six protein expression levels weighted by coefficients that were predefined on the basis of ROC analysis of the TCGA cohort (see Methods for details).

### Association of PSES with surgical stage in patients with EEC

**Figure 2:**
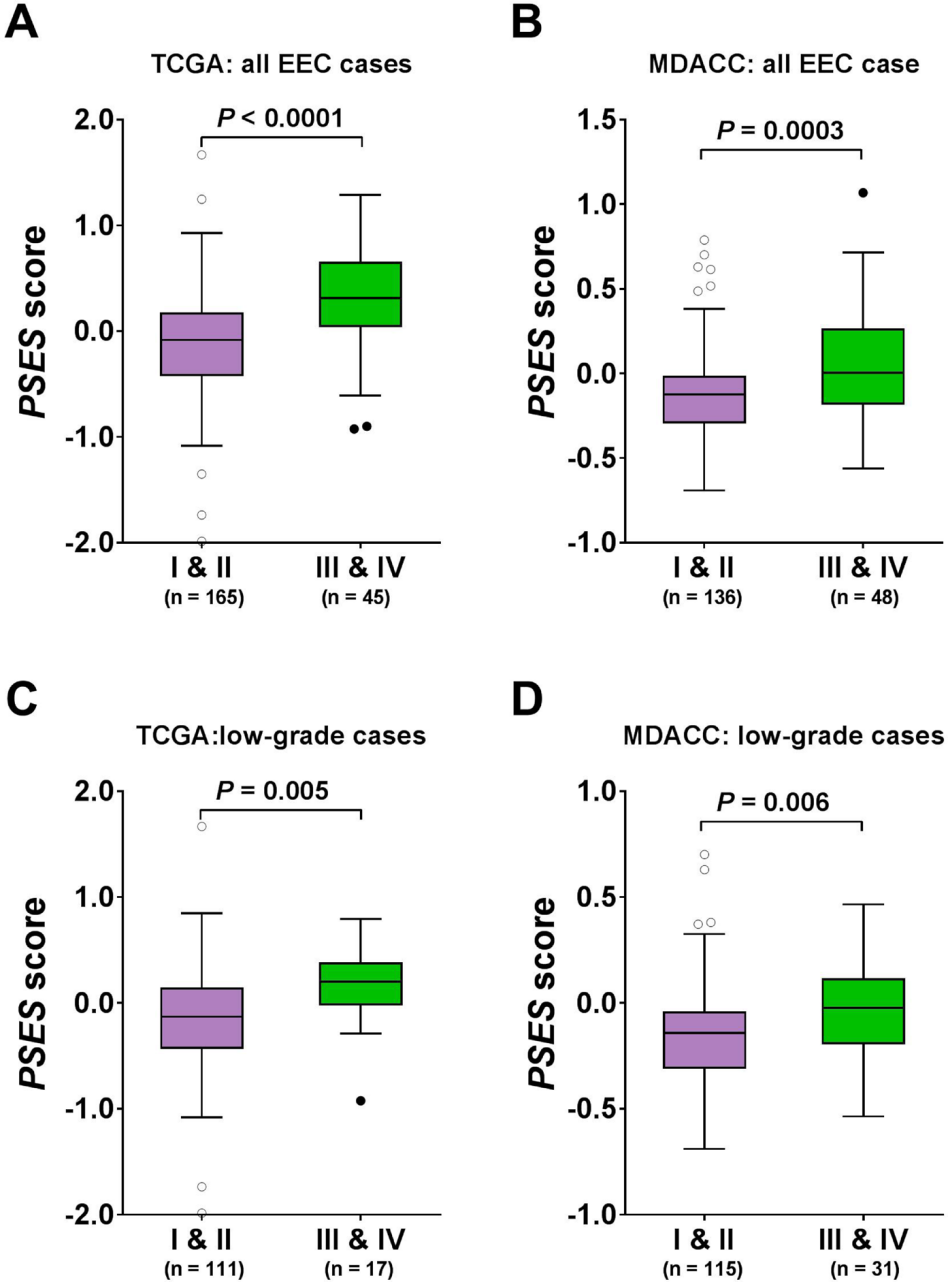
Association of PSES with tumor stage in patients with EEC (**A**) Box plots represent PSES scores in EEC samples from patients with stage I & II disease (*n* = 165) and from patients with stage III & IV disease (*n* = 45) in the TCGA cohort. The central line of each box is the median value, and the edges are the 25th and 75th percentiles. The whiskers extend to the 10th and 90th percentiles, and data points outside the whiskers are plotted individually as circles or dots. *P* values were calculated using two-sided Mann–Whitney test. (**B**) The predictive performance of PSES score was validated in an independent MDACC data set. PSES scores were significantly higher in patients with stage III & IV disease in the MDACC cohort (*P* = 0.0003, Mann–Whitney test). Among the low-grade (grade 1 & 2) EEC patients, tumors with advanced stage disease had statistically significantly higher PSES scores than did those with early stage disease in both (**C**) the TCGA cohort (*n* = 128, *P* = 0.0046, Mann–Whitney test) and (**D**) the MDACC cohort (*n* = 146, *P* = 0.0055, Mann–Whitney test).

We calculated PSES scores for each sample in the TCGA cohort. Values ranged from -1.9865 to 1.6682 across the 210 samples, of which 104 had positive PSES scores and 106 had negative scores. PSES scores were significantly higher in patients with advanced stage disease than in patients with early stage disease (*P* < 0.0001, Mann–Whitney test) (Figure 2A). To examine the impact of tumor purities on this result, we carried out two additional analyses. First, we compared tumor purities between early-stage and advanced-stage disease and found there was no significant difference in these two groups of patients (*P* = 0.4459, Supplementary Figure 2A). Secondly, we calculated the purity adjusted PSES score by dividing the original PSES score by tumor purity, and the purity-adjusted PSES score remained significant correlation with patient surgical stage (*P* = 0.0002, Supplementary Figure 2B). Note that the statistical significance was slightly compromised likely because the number of analyzed samples becomes smaller when tumor purity data were incorporated into this analysis. In addition, we obtained the percentage of stroma, lymphocyte, macrophage, and neutrophil cells in the EEC tissue specimen and found that PSES score was not significantly correlated with these cellular compositions (Supplementary Figure 3). Collectively, these data indicate that PSES is significantly correlated with surgical stage, independent upon tumor purities. We next validated PSES in an independent patient cohort and performed RPPA profiling on an additional set of 184 EEC cases that were not included in the TCGA cohort. PSES score for each sample was computed in a similar manner in the validation set. Patients with advanced stage disease demonstrated significantly higher PSES scores than those with early stage disease (*P* = 0.0003, Mann–Whitney test) (Figure 2B), which was consistent with the results in the TCGA cohort. Because low-grade (Grade 1 or 2) tumors are less likely to have advanced stage disease, it is clinically of interest to be able to predict advanced stage tumors among patients with low-grade EEC. Prominently, among patients with grade 1 or 2 tumors, those with advanced stage disease had statistically significantly higher PSES scores than did those with early stage disease in both the TCGA dataset (*P* = 0.0046, Mann–Whitney test) (Figure 2C) and the MDACC dataset (*P* = 0.0055) (Figure 2D). Taken together, these results demonstrated that PSES is significantly correlated with tumor surgical stage even among patients with low-grade disease, as evidenced by an external validation.

To test whether PSES is an independent predictor of EEC patients with advanced stage disease, we further preformed multivariate logistic regression analyses. Even after adjusting for patients’ age, grade, vital status, or recurrent disease status, the odds ratios for patients with high PSES scores were 4.64 (95% CI = 2.06 to 10.48, *P* = 2.2E-04) in the TCGA cohort and 2.94 (95% CI = 1.23 to 7.00, *P* = 0.015) in the MDACC cohort (Table 2). In addition, we calculated the sensitivity and specificity for each of the individual variables to predict EEC patients with advanced stage disease (Supplementary Table 1). As compared to the other predictors, PSES improves the predictive sensitivity very much. We next correlated the PSES score with gene mutations frequently observed in endometrial cancer (Supplementary Figure 4). Patients with *TP53* mutation had significantly higher PSES scores than those with *TP53* wide-type. No significant correlation was observed between PSES scores and *PTEN* mutation or *CTNNB1* mutation.

**Table 2:** Multivariate logistic analyses for PSES scores and various diagnostic factors in patients with EEC

	Variables^†^	OR (95% CI)	*P*
**TCGA**	*Advanced stage vs early stage tumors*		
	PSES score	3.96 (1.80 to 8.73)	.001
	Age, >60 years vs ≤60 years	0.53 (0.24 to 1.17)	.117
	Grade, Gr3 vs Gr1/2	2.33 (1.06 to 5.12)	.036
	Vital status, deceased vs living	1.87 (0.50 to 7.02)	.355
	Recurrence, yes vs no	4.72 (1.88 to 11.86)	.001
**MDACC**	*Advanced stage vs early stage tumors*		
	PSES score	5.37 (1.27 to 22.65)	.022
	Age, >60 years vs ≤60 years	1.74 (0.70 to 4.32)	.228
	Grade, Gr3 vs Gr1/2	0.92 (0.31 to 2.76)	.882
	Vital status, deceased vs living	1.32 (0.30 to 5.92)	.712
	Recurrence, yes vs no	10.44 (3.55 to 30.73)	<.001

Abbreviations: CI, confidence interval; OR, odds ratio.

^†^PSES score was treated as a continuous variable and all other covariates were binary: age (0 for an age of 60 years or less and 1 for an age of greater than 60 years), grade (0 for a grade of 1 or 2 and 1 for a grade of 3); vital status (0 for living and 1 for deceased), and recurrence (0 for a tumor with no recurrence and 1 for a tumor with recurrence).

### Association of PSES with patient outcomes

**Figure 3:**
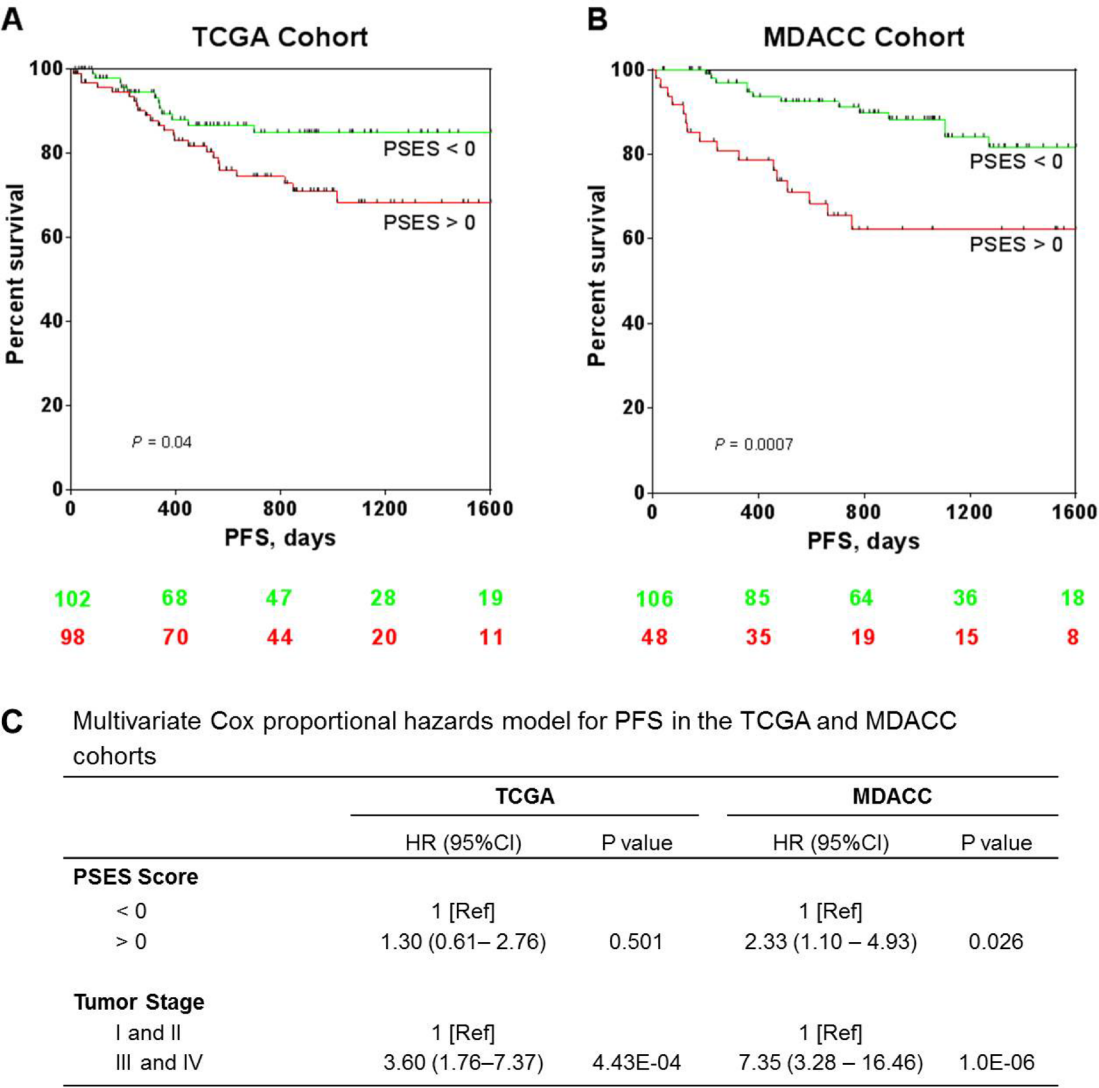
Association of PSES with tumor prognosis in patients with EEC (**A**) The PFS rate in EEC patients with positive PSES scores (*n* = 98) was statistically significantly lower than that for patients with negative PSES scores (*n* = 102) (*P* = 0.04, log-rank test) in the TCGA cohort. (**B**) The PFS rate in EEC patients with positive PSES scores (*n* = 48) was statistically significantly lower than that for those with negative PESE scores (*n* = 106) (*P* = 0.0007, log-rank test) in the MDACC cohort. Patients with missing survival data were excluded from this analysis. The numbers of patients at risk at various time points are shown below each curve. (**C**) Multivariate Cox proportional hazards model analysis of PFS with PSES score and patient tumor stage as covariates in both the TCGA and MDACC cohorts.

While PSES was generated on the basis of tumor stage, we additionally performed Kaplan-Meier survival analysis to assess the capacity of PSES to differentiate patients by PFS. Patients with positive PSES scores had statistically significantly worse PFS in both the TCGA cohort (*P* = 0.04, log-rank test) (Figure 3A) and the MDACC cohort (*P* = 0.0007, log-rank test) (Figure 3B). To test whether this result was independent of tumor stage, we performed two different types of analyses. First, we added both PSES score and tumor stage as covariates to a Cox proportional hazards model and estimated the hazard ratio statistic. Although stage remained a strong predictor in both cohorts, positive PSES score was significantly associated with shorter PFS in the MDACC cohort even after adjustment by tumor stage (HR, 2.33, 95% CI = 1.10 to 4.93, *P* = 0.026) (Figure 3C). Secondly, we stratified patients into either early-stage or advanced-stage group and performed Kaplan-Meier survival analysis using the PSES scores separately in these two groups. PSES score was significantly associated with PFS in either group in the MDACC cohort but not in the TCGA cohort (Supplementary Figure 5). Taken together, these data suggested that PSES score is significantly correlated with patient PFS, independent of surgical stage. Although patients with higher PSES score appeared to have worse overall survival, this trend was not statistically significant (Supplementary Figure 6).

### Biological Interpretation of PSES

**Figure 4:**
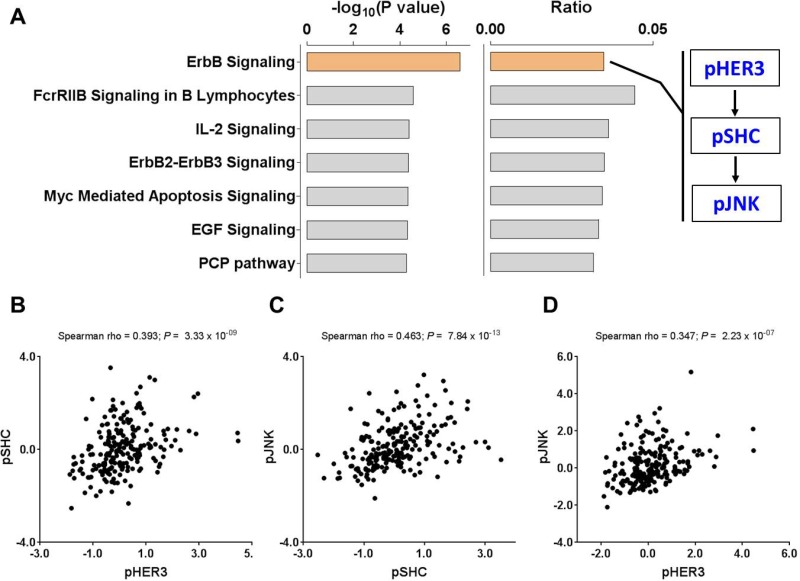
Biological interpretation of PSES (**A**) Significantly enriched pathways in the four differentially expressed proteins used for construction of PSES where cartoon of ErbB signaling pathway involving the three phosphorylated proteins is also shown. The right plot on this panel shows the ratio of genes in the PSES proteins to all the genes included in the signaling pathway. (**B–D**) Expression correlation of the three proteins that are involved in the ErbB signaling pathway where (B) pHER3 vs pSHC, (C) pSHC vs pJNK and (D) pHER3 vs pJNK.

To characterize the biological properties of PSES, we used publicly available tools to associate the predictive proteins with gene ontology (using the GO database) [16] as well as pathway annotation (Ingenuity Pathway Analysis, https://www.qiagenbioinformatics.com/products/ingenuity-pathway-analysis/). The Ingenuity Knowledge Base including all proteins was used as a reference set and the statistical significance was determined by the Fisher's exact test. We focused the analyses on the four differentially expressed proteins/phosphoproteins. Dvl3 is a binding protein that controls signal transduction activity in the Wnt pathway and that all three phosphorylated proteins (pHER3, pSHC, and pJNK) are involved in kinase signaling through cell surface receptors (Supplementary Table 2). Pathway analysis showed that ErbB signaling is most significantly enriched in the PSES proteins (*P* = 5.1E-05) (Figure 4A). This pathway is associated with tumor angiogenesis and involves the three phosphorylated proteins that were significantly downregulated in advanced stage tumors (Supplementary Figure 7). Interestingly, a higher frequency of ErbB expression and activation is present in ductal carcinoma *in situ* than in invasive breast cancer. Canonically, Shc1, a docking partner of ErbB receptors [17], regulates the JNK signaling pathway [18]. Moreover, the expression levels of these three phosphoproteins were strongly and significantly correlated with one another, further suggesting significant inactivation of this signaling pathway in advanced stage EEC tumors (Figure 4B).

## DISCUSSION

Using RPPA profiling of nearly 400 EEC samples, the largest cohort with protein data yet reported, in this study we developed a predictive scheme that successfully distinguished patients with advanced stage disease from those with early stage disease, independent of patient's age, tumor grade, vital status, or recurrence status. We further demonstrated that PSES has both prognostic value and biological interest. The clinical stage determined at the initial diagnosis is typically based on the results of a physical exam, biopsy, and imaging tests before surgery. The pathologic stage is determined much later by examining tissue removed during an operation, and thus gives the health care team more precise information that can be used to predict treatment response and outcome. One potential use of the PSES algorithm would be in analysis of preoperative EEC tumor biopsies obtained during initial diagnosis that could then be used to direct the appropriate surgical approach. Specifically, our PSES score could help select for patients who should undergo more extensive lymph node dissections at the time of hysterectomy [19]. Given its strong correlation with pathological stage, the clinical stage information should be incorporated into the PSES prediction scheme in order to improve the predictive sensitivity and specificity. A protein-driven model that can be evaluated using immunohistochemistry may be more practical and accurate in clinical management [20], while also being less susceptible to expression variations of individual proteins. Different from other predictors such as histologic grade, patient age, or even clinical stage, the PSES predictor projects treatment strategies and likely facilitates identification of novel targets for therapeutic intervention.

Feature selection based on statistical comparison is dependent upon sample size and patient characteristics. For example, the gene sets discovered to be associated with platinum-based chemotherapy resistance exhibited a wide range of 14 to 1,727 genes from several ovarian cancer studies (sample sizes of 6 to 119), and only seven genes were observed as overlapping [21]. To capture these factors, we sought to identify informative features that exhibited statistically significant differences on average in 500 different training sets that were randomly selected from the TCGA cohort and which comprised different numbers of samples. A predictive model consisting of the thus-selected features was robust to tumor heterogeneity and sample size, as demonstrated by validation in an independent cohort.

PSES also offers biological insight into endometrial tumor progression. Interestingly, the differentially expressed proteins are significantly enriched in the ErbB signaling pathway, which involves the three phosphoproteins (pHER3, pSHC, pJNK) that were significantly downregulated in advanced stage EEC tumors. Strong expression correlation among these phosphoproteins further supported an abnormality in this signaling pathway. Dysregulation of these proteins or related pathways in the role of tumor suppression or in disease with favorable prognosis has previously been demonstrated in several other cancers. Activation of the JNK signaling pathway was previously reported to contribute to apoptosis and growth inhibition in human hepatoblastoma [22] and osteosarcoma [23] cells. In contrast, JNK deficiency significantly increases tumor formation in breast cancer [24]. Compared with other molecular subtypes, luminal A/B breast cancer, which has a relatively favorable prognosis, exhibits higher ERBB3 expression [25]. Likewise, low expression level of p66, an isoform of pSHC, was recently reported to be significantly correlated with worse survival rate in lung cancer [26]. Collectively, these findings are biologically consistent with the downregulation of the ErbB signaling pathway in advanced stage tumors in the setting of EEC. Consistently, higher expression levels of the EGFR protein (a member of the ErbB family) and p38 MAPK phosphoprotein (a member of the MAPK family) were previously reported to be associated with a good prognosis in early-stage EEC [27].

On the other hand, the disheveled segment polarity protein 3 (encoded by *DVL3*), a key mediator of Wnt/β-catenin signaling [28], which was significantly upregulated in the advanced stage EEC tumors, has been shown to be a driver of lung cancer metastases [29, 30]. Tumors with a relatively lower level of Dvl3 were correlated with improved sensitization to IGF1R inhibition and longer PFS in patients treated with IGF1R antibodies [31]. The Wnt/β-catenin signaling pathway appears to be a predominant factor driving tumor progression in endometrial cancer, though mediators for the pathway activation are different from low-grade to high-grade EEC. The Wnt pathway is activated either by β-catenin exon 3 mutations in low-grade endometrioid carcinoma [7] or by overexpression of Dvl3 protein in high-grade endometrioid carcinoma.

It was previously reported that two randomized clinical trials demonstrated different effects of adjuvant chemotherapy on PFS [32]. In striking contrast to a lack of effect in the MaNGO trial, which recruited over 99% Stage IIB-IIIC patients, the NSGO/EORTC trial, which consisted of 98% Stage IA-IIA patients, showed a favorable effect of chemotherapy on PFS. These results suggest that benefit from adjuvant chemotherapy may reside largely within the cohort of patients with stage I-II tumors, which could be reliably predicted by PSES in this study. Nonetheless, the association of PSES with stage should be validated in a prospectively acquired EEC cohort before we could translate it into routine clinical practice.

In summary, PSES is a useful prognostic factor and offers potential targets for therapeutics. The finding has clinical implications for risk assessment and early intervention for patients with endometrial cancer.

## MATERIALS AND METHODS

### Patient samples

Protein expression profiling and clinicopathologic annotation for 210 EEC cases were obtained from the TCGA data portal [6] on March 1, 2013. Patients underwent surgical resection but received no prior treatment for their disease. Clinicopathologic diagnoses were made at local tissue source sites and further confirmed by TCGA. Cases were reviewed and pathologically staged according to the 2009 FIGO staging system into 4 major sub-stage divisions (stage I, II, III, and IV) [6]. As a validation cohort, an additional 184 EEC cases were diagnosed at MDACC from 1998 to 2009, and the tumor samples were reviewed for grade and stage by two independent pathologists. The study was approved by the National Cancer Institute (training set) and by the institutional review board at MDACC (validation set). Detailed patient demographic and clinicopathologic characteristics for both cohorts are described and compared in Table 1.

### RPPA Profiling

Reverse-phase protein array (RPPA) is a high-throughput antibody-based technique for simultaneously measuring protein expression levels in a large number of biological samples [33]. Quantitative protein expression profiles in the training set were obtained from TCGA [6]. The 210 TCGA EEC samples included in this study were those who had both RPPA data and clinical annotation. RPPA profiling of samples in the validation set (MDACC cohort) was measured and pre-processed, including sample-wise median center and log 2 transformation, at the MD Anderson RPPA core facility using standard operating procedures and validated antibodies [33]. For the purpose of comparison between TCGA (170 antibodies) and MDACC (187 antibodies) data sets, a total of 138 antibodies that were in common across these two cohorts were selected for all downstream analyses.

### Identification of predictive proteins

The overall flow chart of the study design shown in Supplementary Figure 8 summarizes the procedure used to construct and validate the protein-based scoring scheme for predicting clinical stage of EEC patients. The predictive proteins (features) were first identified to be differentially expressed via supervised analysis of the advanced versus early stage tumors in the TCGA set. Firstly, we randomly selected a subset of patients from the TCGA cohort with a set size ranging from 30 to 210. Then, we performed supervised analysis to identify differentially expressed proteins/phosphoproteins between advanced stage versus early stage tumors within this subset and calculated expression (fold-change in term of log2 ratio) and statistical (*P* value) differences for all proteins and phosphoproteins. We repeated this process 500 times (Supplementary Figure 9). For each protein, the fold-changes and *P* values varied with subsets (Supplementary Figure 10), presumably because different subsets had different sample sizes and different patient characteristics. We then calculated the arithmetic mean of the fold-changes and geometric mean of the *P* values for each protein from all 500 randomly selected subsets. Using an average *P* value cutoff of 0.05, we identified the predictive protein to use in developing the prediction algorithm. EEC tumors are characterized by expression of estrogen receptor alpha (ER) and progesterone receptor (PR); hence, we included these two proteins in the predictive model to account for baseline fluctuation on other clinicopathological variables such as age, grade, and recurrence status.

### Construction of predictive model

The predictive model was constructed using a weighted voting algorithm [34, 35]. The protein scoring of EEC staging (PSES) of the j^th^ sample is defined as

$${\text{PSES}}_{j}=\sum_{i=1}^{N}\omega_{i}•\left(x_{\text{ij}}-\mu_{i}\right)$$

where N is the number of predictive proteins (6 in this case); j represents samples (j = 1,2,…); i represents proteins with non-zero weights (i = 1,2, …,6); ω_i_ is the weighting factor associated with the i^th^ protein; x_ij_ is the expression level of the i^th^ protein for the j^th^ patient; and μ_i_ represents the average expression of the i^th^ protein across the entire cohort. The weighting factors reflect the contribution of proteins to the predictive model and are first initialized to their relative expressions (rx) [36] between the advanced stage and early stage groups:

$$\omega_{i}=\frac{{\text{rx}}_{i}}{\sum_{i=1}^{N}\left|{\text{rx}}_{i}\right|}$$

$${\text{rx}}_{i}=\frac{\mu_{i,\text{hi}}-\mu_{i,\text{lo}}}{\sigma_{i,\text{hi}}+\sigma_{i,\text{lo}}}$$

where μ_i,hi_, σ_i,hi_ (μ_i,lo_, σ_i,lo_) represent the average expression and standard deviation of the i^th^ protein in the advanced stage (and early stage) groups, respectively.

To reduce the potential of overfitting [37], the initial weighting factors were next subjected to unconstrained nonlinear optimization using a derivative-free method [38]. The genetic algorithm was employed in Matlab, and refined using *fminsearch*. *fminsearch* starts at the initial weighting vector, *x_0_* = [ω_1_, ω_2_, ω_3_, w_4_, w_5_, w_6_] and finds a local minimum of the custom-defined function described in *Fun(x)*.

$$\hat{x}=\text{fminsearch}\left[\text{Fun}(x),\;x_{0}\right]$$

$$\text{Fun}(x)=1-\text{ROC }({\text{PSES}}_{\text{hi}}^{\left(x\right)},{\text{PSES}}_{\text{lo}}^{\left(x\right)})$$

$$\hat{x}=\;\left[{\hat{\omega }}_{1},\;{\hat{\omega }}_{2},\;{\hat{\omega }}_{3},\;{\hat{\omega }}_{4},\;{\hat{\omega }}_{5},\;{\hat{\omega }}_{6}\right]$$

where *PSES_hi_^(x)^* and *PSES_lo_^(x)^* are the PSES scores for advanced and early stage tumors respectively and depend upon the weighting factor, *x*. ROC denotes the ROC curve analysis of these two scores; the corresponding value of the area under the curve (AUC) for each *x* was then calculated. Minimizing *Fun(x)* is equivalent to maximizing the predictive performance assessed by receiver operating characteristics (ROC) curve analysis. The *x* hat represents the optimized weighting factor vector that gives rise to a maximum AUC value. The ROC curves evaluated via PSES scores were calculated as examples at the initialization and at the end of optimization (Supplementary Fgure 11).

The PSES score was calculated for each of the 210 TCGA samples as the sum of the protein expression levels multiplied by the optimized weighting factors,

$${\text{PSES}}_{j}=\sum_{i=1}^{N}\;{\hat{\omega }}_{i}•\left(x_{\text{ij}}-\mu_{i}\right)$$

Specifically, PSES was defined as follows: 0.3597 × Dvl3–0.1874 × JNK-pT183_pT185–0.017 × Shc_pY317–0.1976 × HER3_pY1298–0.06 × ER-alpha–0.0678 × PR, where protein expressions were mean centered. A plus or minus sign indicates that increased expression is associated with tumors with either advanced or early stage disease.

### Statistical analysis

Mann–Whitney analysis of variance was used to evaluate the statistical differences in protein levels and PSES scores between early and advanced stage tumors. The Spearman correlation test was used to examine expression correlation among the three identified phosphorylated proteins. The Youden index [39] was used to determine the optimal PSES cutoff value in distinguishing patients with advanced and early stage disease. A multivariable logistic regression model was used to calculate the odds ratios (ORs) for clinicopathological variables associated with advanced stage tumors according to the PSES scores.

Survival analyses were performed using the Kaplan-Meier method, and the difference in survival was examined with the use of log-rank tests. Overall survival (OS) was defined as the interval from the date of initial surgical resection to the date of last known contact (censored) or death. Progression-free survival (PFS) was defined as the interval from the date of initial surgical resection to the date of progression, recurrence, or last known contact (censored). Statistical significance was defined as *P* < 0.05, and all tests were two-sided. Analyses were primarily performed using the scientific software Matlab, version 8.4 (MathWorks, Inc., Natick, MA, USA), SPSS version 18 (SPSS Inc., Chicago, IL, USA), and GraphPad Prism, version 6 (GraphPad Software, Inc., La Jolla, CA, USA).

## SUPPLEMENTARY MATERIALS FIGURES AND TABLES


