# Supplementary material for: Predicting high-risk endometrioid carcinomas using proteins
Source: Oncotarget. 2018 Apr 13;9(28):19704–15. doi: 10.18632/oncotarget.24803 (PMC5929419; doi:10.18632/oncotarget.24803)
Supplement: Supplementary file 1 [file oncotarget-09-19704-s001.pdf]

Predicting high-risk endometrioid carcinomas using proteins

SUPPLEMENTARY MATERIALS

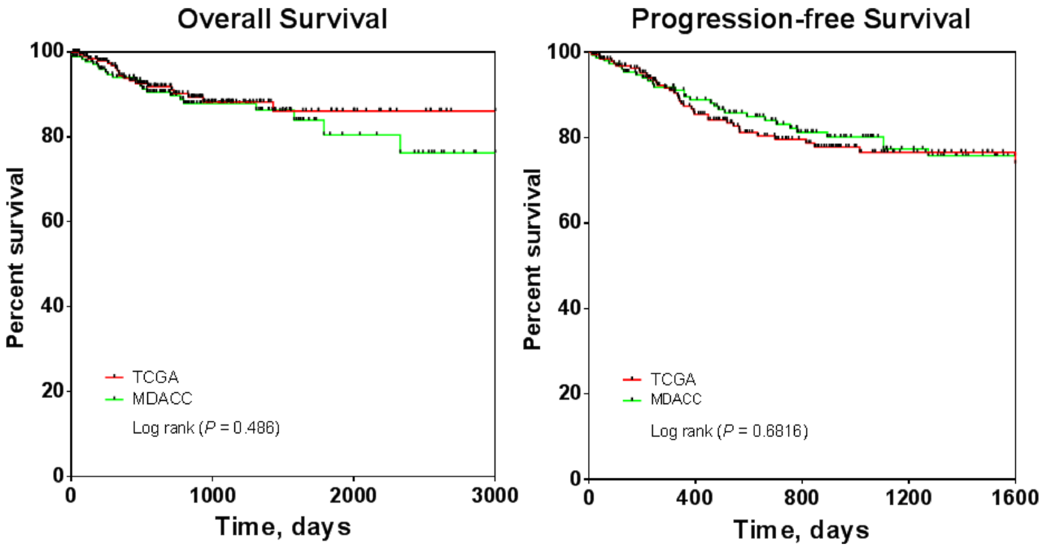

Supplementary Figure 1: Comparison of patient survival between the TCGA and MDACC cohorts. Kaplan-Meier curves of overall survival (left) and progression-free survival (right) between patients in the TCGA and MDACC cohorts. Statistical significance was assessed with the use of log-rank test.

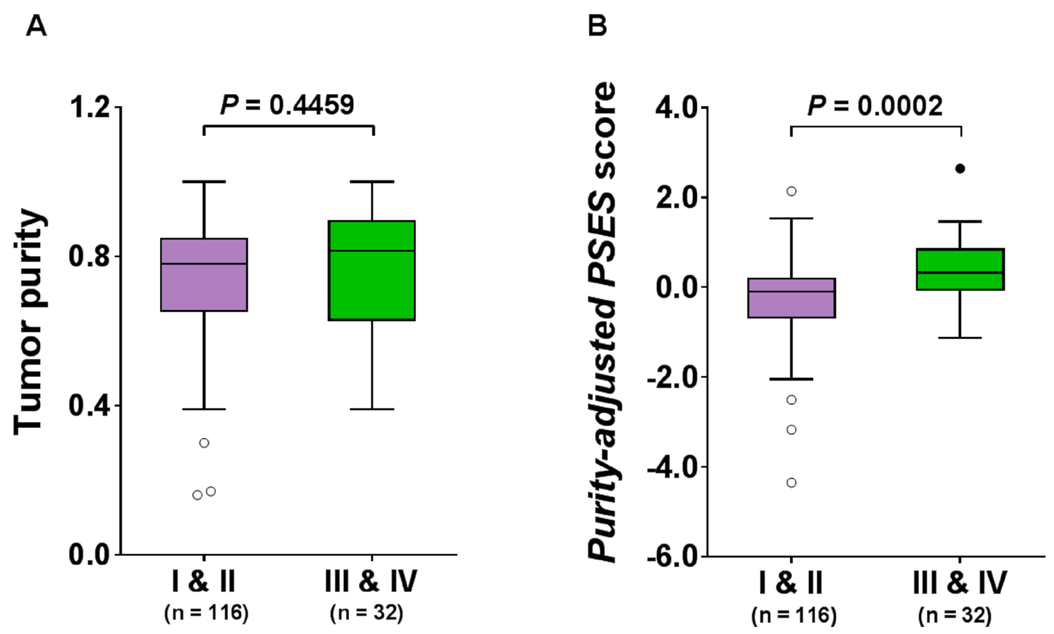

Supplementary Figure 2: (**A**) Correlation of tumor purity with surgical stage. (**B**) Correlation of purity-adjusted PSES scores with surgical stage. Note that the statistical significance is slightly compromised likely because the number of analyzed samples becomes smaller when tumor purity data were incorporated into this analysis.

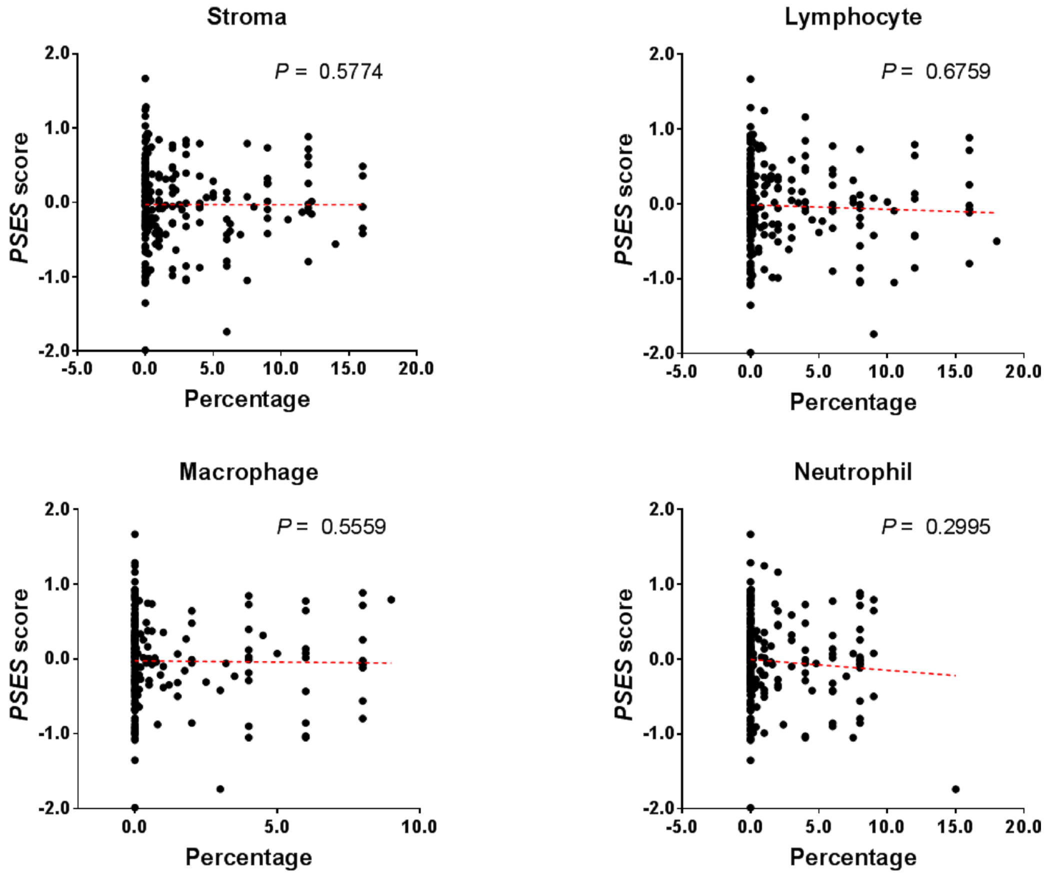

Supplementary Figure 3: Correlation of PSES score with percentage of stroma, lymphocytes, macrophages, and neutrophils cells (estimated from the histology images by the TCGA network pathologists, see TCGA network paper, TCGA, *Nature*. 2013; 497:67–73).

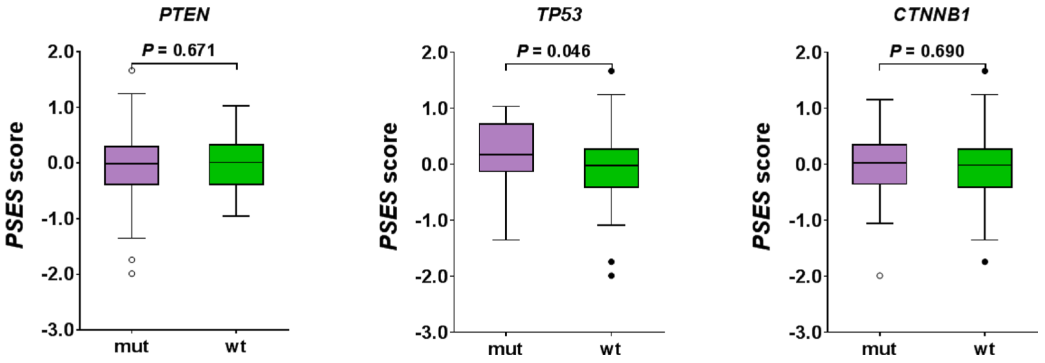

Supplementary Figure 4: Correlation of PSES score with gene mutations commonly observed in endometrial cancer.

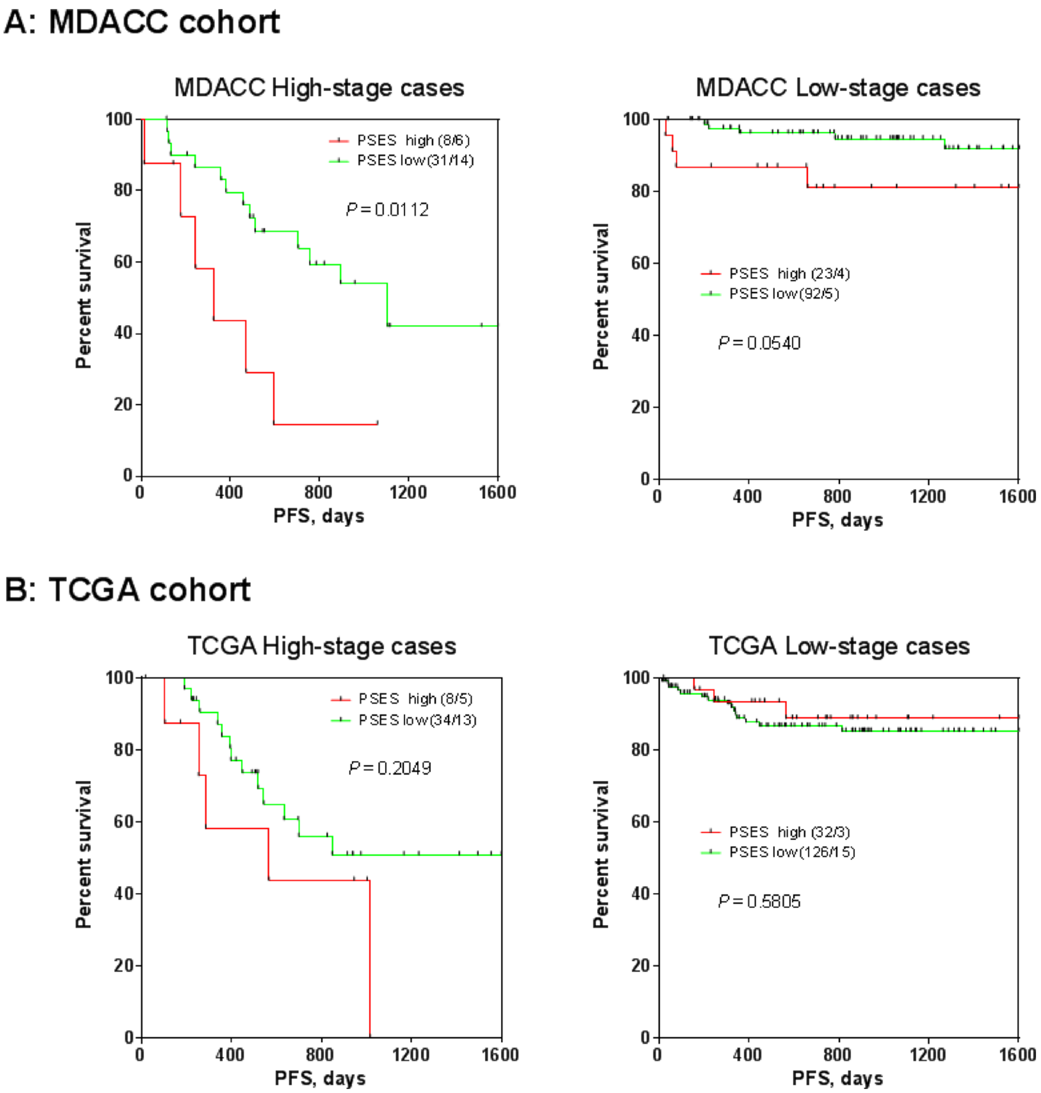

Supplementary Figure 5: Kaplan-Meier analyses of progression-free survival between EEC patients stratified on the basis of PSES scores in either advanced- or early- stage tumors (**A**) in the MDACC cohort and (**B**) in the TCGA cohort.

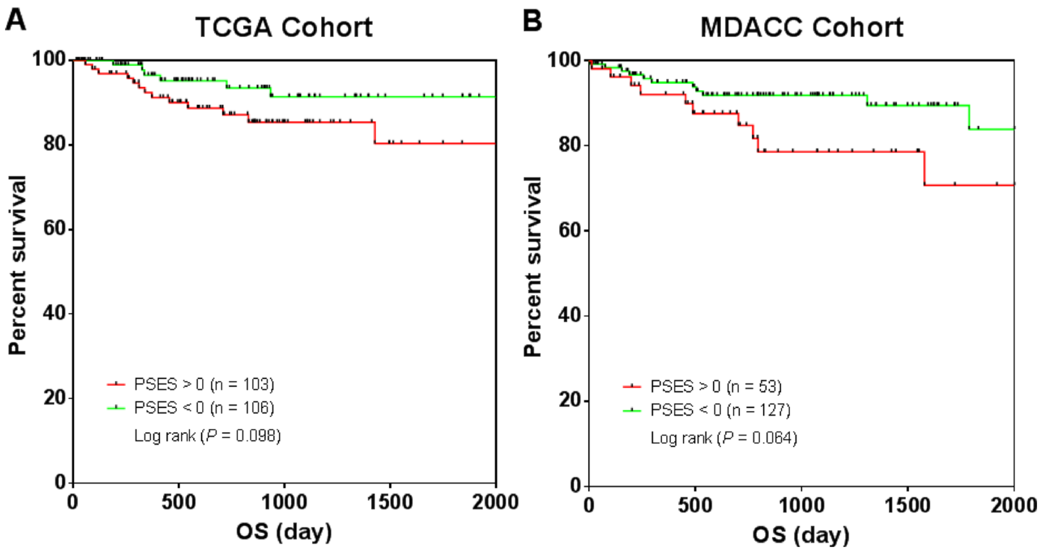

Supplementary Figure 6: Kaplan-Meier analyses of overall survival between EEC patients stratified on the basis of PSES scores (**A**) in the TCGA cohort and (**B**) in the MDACC cohort.

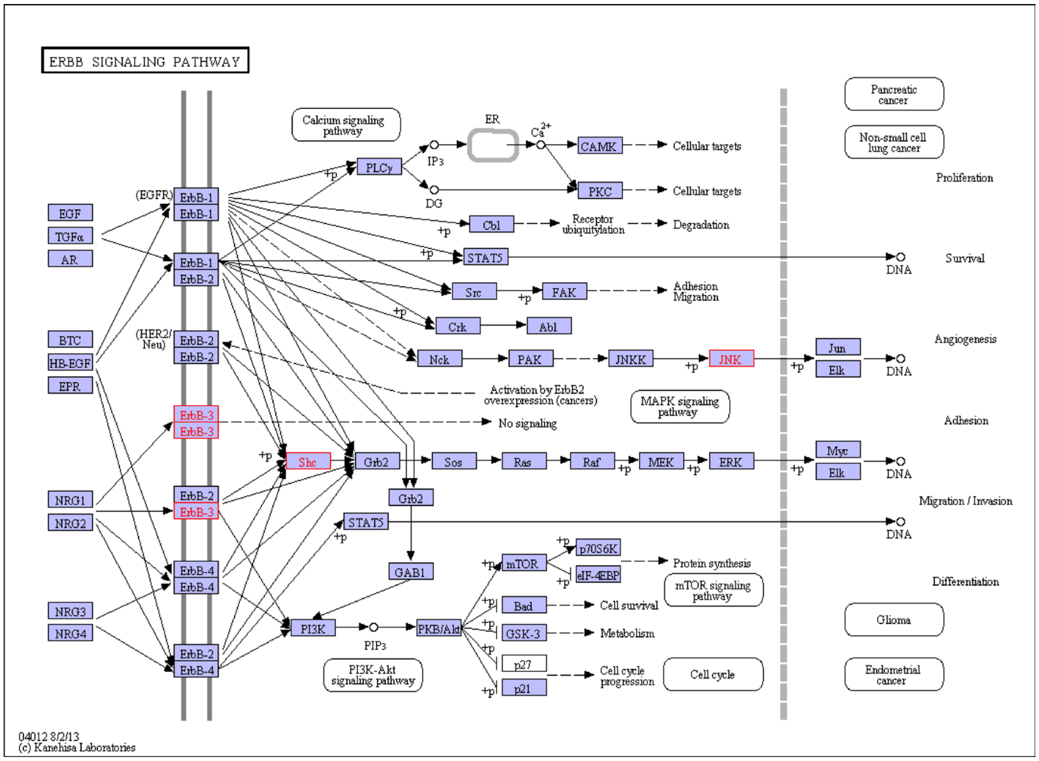

Supplementary Figure 7: The three down-regulated proteins in the advanced-stage tumors (pJNK, pSHC and pHER3) are all involved in the ErbB signal pathway from the KEGG database.

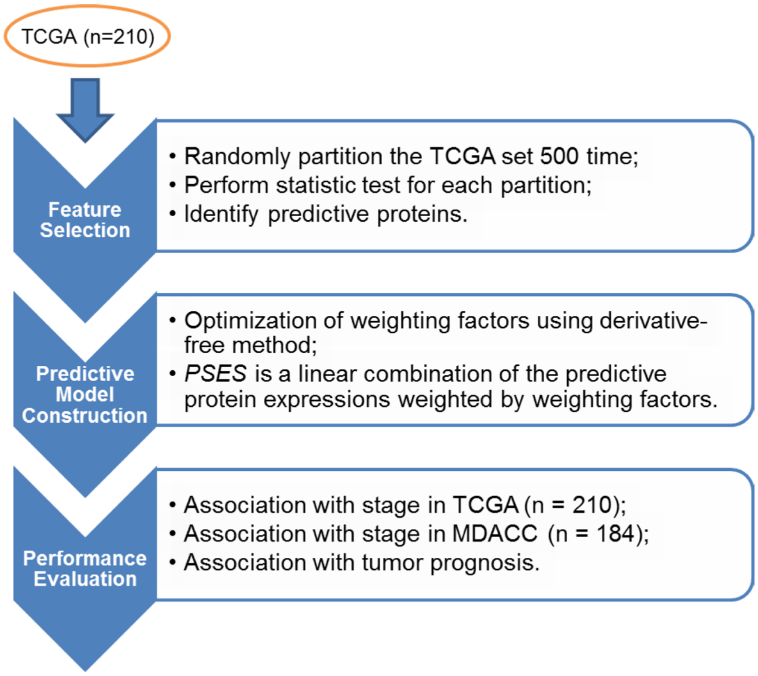

Supplementary Figure 8: Overview of the study design.

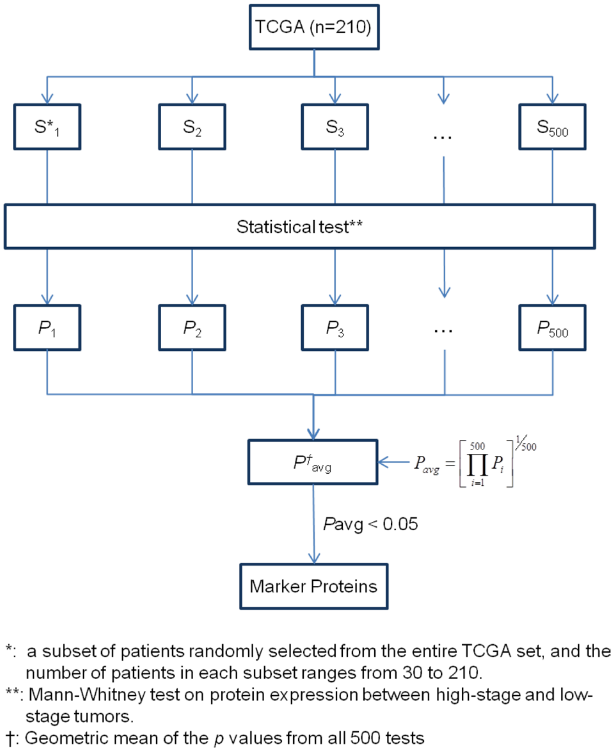

$$P_{avg} = \left[\prod_{i=1}^{500} P_i\right]^{1/500}$$

Supplementary Figure 9: Identification of differentially expressed proteins between advanced-stage and early-stage tumors in the TCGA cohort.

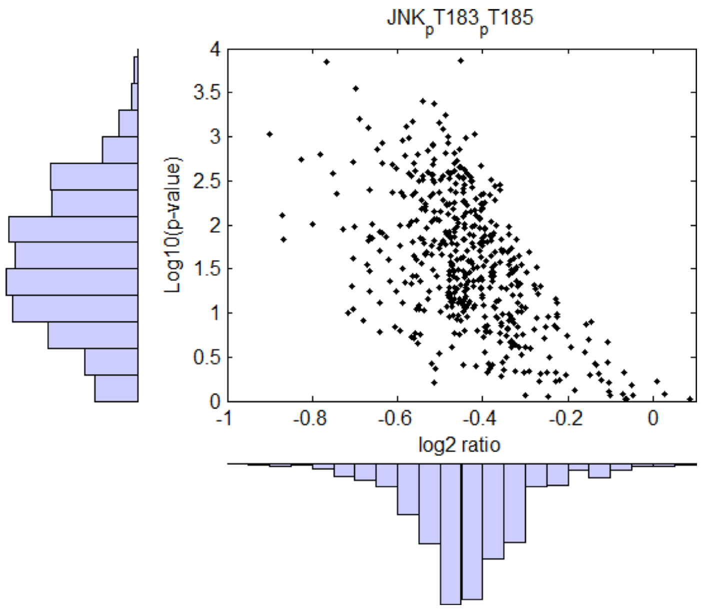

Supplementary Figure 10: The statistical significance and log2 ratio of JNK-pT183-pT185 protein between early and advanced stage tumors calculated from the 500 randomly selected subsets. The histograms show the distribution of these two parameters and each dot represents an individual subset.

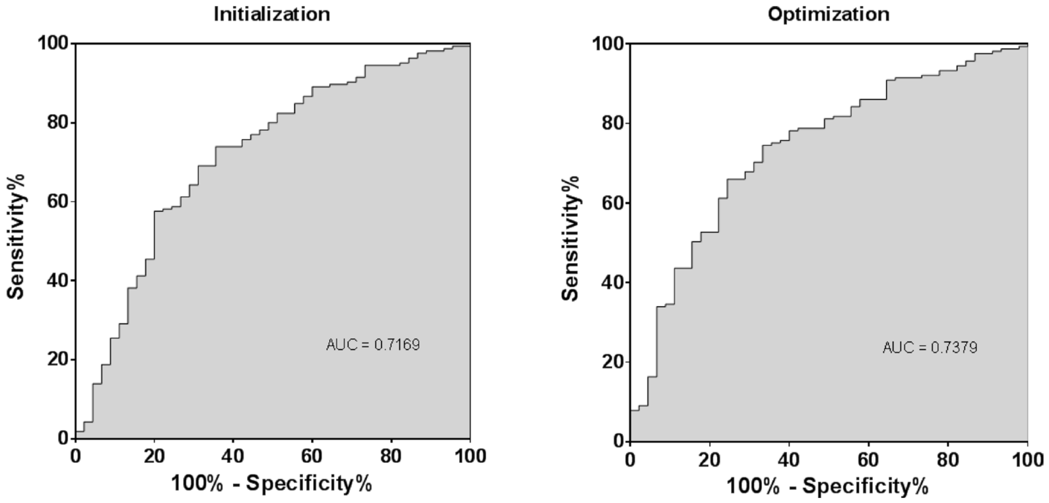

Supplementary Figure 11: The example ROC curves evaluated via PSES scores at the initialization and at end of optimization.

Supplementary Table 1: Sensitivity and specificity for each of individual variables to predict EEC surgical stage in both TCGA and MDACC cohorts

	TCGA		MDACC	
**Variables**	**Sensitivity, %**	**Specificity, %**	**Sensitivity, %**	**Specificity, %**
PSES‡	76	66	58	75
Age, >60 years vs ≤60 years	49	43	64	51
Grade, Gr3 vs Gr1/2	62	67	38	84
Vital status, deceased vs living	22	94	23	92
Recurrence, yes vs no	43	87	58	91

‡The PSES cutoff was derived by receiver operating characteristic curves with Youden's index.

Supplementary Table 2: Gene oncology (GO) ID and term involving each of the four differentially expressed proteins

**Proteins**	**GO *ID***	**GO term**
**Dvl3**		
	GO: 0002020	protease binding
	GO: 0004871	signal transducer activity
	GO: 0005102	receptor binding
	GO:0005109	frizzled binding
	GO:0005515	protein binding
**JNK_pT183_pT185**		
	GO: 0004672	protein kinase activity
	GO: 0004674	protein serine/threonine kinase activity
	GO: 0004705	JUN kinase activity
	GO: 0004707	MAP kinase activity
	GO: 0003824	catalytic activity
**Shc_pY317**		
	GO: 0004713	protein tyrosine kinase activity
	GO: 0005068	transmembrane receptor protein tyrosine kinase adaptor activity
	GO: 0005154	epidermal growth factor receptor binding
	GO: 0005158	insulin receptor binding
	GO: 0005159	insulin-like growth factor receptor binding
**HER3_pY1298**		
	GO: 0004672	protein kinase activity
	GO: 0004713	protein tyrosine kinase activity
	GO: 0004714	transmembrane receptor protein tyrosine kinase activity
	GO: 0004716	receptor signaling protein tyrosine kinase activity
	GO: 0004888	transmembrane signaling receptor activity
